# Quality assurance for MRI-only radiation therapy: A voxel-wise population-based methodology for image and dose assessment of synthetic CT generation methods

**DOI:** 10.3389/fonc.2022.968689

**Published:** 2022-10-10

**Authors:** Hilda Chourak, Anaïs Barateau, Safaa Tahri, Capucine Cadin, Caroline Lafond, Jean-Claude Nunes, Adrien Boue-Rafle, Mathias Perazzi, Peter B. Greer, Jason Dowling, Renaud de Crevoisier, Oscar Acosta

**Affiliations:** ^1^ University of Rennes, CLCC Eugène Marquis, INSERM, LTSI - UMR 1099, Rennes, France; ^2^ The Australian eHealth Research Centre, Commonwealth Scientific and Industrial Research Organisation (CSIRO), Health and Biosecurity, Brisbane, QLD, Australia; ^3^ School of Mathematical and Physical Sciences, University of Newcastle, Newcastle, NSW, Australia; ^4^ Radiation Oncology, Calvary Mater Newcastle Hospital, Newcastle, NSW, Australia

**Keywords:** quality assurance, voxel-wise analysis, population-based evaluation, synthetic CT assessment, dosimetric assessment, MRI-only radiation therapy

## Abstract

The quality assurance of synthetic CT (sCT) is crucial for safe clinical transfer to an MRI-only radiotherapy planning workflow. The aim of this work is to propose a population-based process assessing local errors in the generation of sCTs and their impact on dose distribution. For the analysis to be anatomically meaningful, a customized interpatient registration method brought the population data to the same coordinate system. Then, the voxel-based process was applied on two sCT generation methods: a bulk-density method and a generative adversarial network. The CT and MRI pairs of 39 patients treated by radiotherapy for prostate cancer were used for sCT generation, and 26 of them with delineated structures were selected for analysis. Voxel-wise errors in sCT compared to CT were assessed for image intensities and dose calculation, and a population-based statistical test was applied to identify the regions where discrepancies were significant. The cumulative histograms of the mean absolute dose error per volume of tissue were computed to give a quantitative indication of the error for each generation method. Accurate interpatient registration was achieved, with mean Dice scores higher than 0.91 for all organs. The proposed method produces three-dimensional maps that precisely show the location of the major discrepancies for both sCT generation methods, highlighting the heterogeneity of image and dose errors for sCT generation methods from MRI across the pelvic anatomy. Hence, this method provides additional information that will assist with both sCT development and quality control for MRI-based planning radiotherapy.

## 1 Introduction

Magnetic resonance imaging (MRI) is becoming increasingly integrated into clinical radiotherapy (RT) planning and monitoring. MRI-guided RT is motivated by the superior soft tissue contrast compared to CT and the non-ionizing modality. However, MRI does not provide information on the electron density of tissue, which is essential for radiotherapy dose calculation. To overcome this issue, several approaches to generate synthetic CT (sCT) in Hounsfield units (HU) from a specific MRI have been developed (1, 2). These include bulk-density (3, 4), atlas-based (5), and machine-learning models, such as patch-based methods with feature extraction (6) and, more recently, deep-learning models (DLMs) (6–12).

Currently, sCT image quality assessment is based on global metrics that measure the discrepancies between reference CT and the corresponding sCT (12, 13). The most common are intensity-based (14) metrics, like the mean absolute error (MAE), mean error (ME), mean squared error (MSE), and peak signal-to-noise ratio (PSNR). Structural similarity (SSIM) (15, 16) is also often computed. These metrics have been reported at a global level, restricted to a single value describing the agreement within the body contour of the patient or within an organ (12). Regarding dosimetric evaluation, the dose distributions obtained from sCT are assessed by comparing the dose–volume histogram (DVH) and gamma analysis (17–20) to the ground truth (dose distribution from reference CT).

DVHs are volume-based statistics that are not relatable to spatial locations, while gamma are spatial distributions; they are usually condensed to a single pass-rate metric, and gamma scores are difficult to interpret clinically. For sCT evaluations, each patient is usually assessed in isolation and the results are then combined. However, it has been reported that errors might appear heterogeneously distributed across different tissue densities (6, 16, 21–24).

Assessing the spatial distribution of errors at a population level may help to identify their origin as well as clinical impact and may subsequently improve the accuracy of sCT generation methods. It can also be useful to compare and select sCT generation methods, and, to a large extent, it may lead to the introduction of quality control protocols within the MRI-based RT planning workflow.

Voxel-wise population analysis can provide powerful tools to assess the clinical impacts of image and dose difference across individuals (25, 26). However, their application requires an accurate non-rigid registration of a whole population to a single coordinate system and the implementation of voxel-wise statistical tests. Previous preliminary work has demonstrated the feasibility of this method, but the analysis methods were limited in clinical scope (27).

The aim of this paper was to propose a multiscale strategy to assess the accuracy of sCT generation methods, starting with a standard error evaluation in the whole pelvis followed by the assessment of organ errors and finally by the implementation of a voxel-wise workflow.

The whole scan population was brought to the same coordinate system *via* a customized non-rigid registration method. Two different sCT generation approaches were chosen as examples to illustrate the methodology: a bulk-density method (BDM) and a deep-learning method, based upon a generative adversarial network (GAN) architecture (6, 28). Then, a comprehensive population-based statistical analysis is performed, including a permutation test adapted to non-parametric paired data and the evaluation of the error dispersion at a voxel-wise scale for each method. The presented methodology not only provides a population spatial quantification of the sCT image value and dose errors but also allows comparison across different sCT generation approaches using the same dataset.

## 2 Materials and methods

### 2.1 Data

A cohort of 39 patients with prostate cancer aged 58–78 years were used to generate sCT scans. For each patient, a CT scan was acquired on a GE LightSpeed RT or a Toshiba Aquilion (256 × 256 × 128 matrix with a voxel size of 1.17 mm × 1.17 mm × 2.5 mm or 2.0 mm), and a T2-weighted MRI was acquired on a Siemens Skyra 3T in the treatment position (resolution of 1.6 mm × 1.6 mm × 1.6 mm). Each CT was resampled and registered to the corresponding MRI *via* a symmetric rigid registration followed by a structure-guided non-rigid method (29, 30) to rectify the main anatomical variations due to the delay between both acquisitions.

MRI was then preprocessed to correct non-uniformity (31) with the Insight Toolkit Library.

As some organs’ delineation, crucial for the interpatient registration, were incomplete, voxel-wise analysis was performed on the 26 patients with bones, prostate, bladder, and rectum delineated on MRI by two physicians. The rectal length started at 2 cm below the clinical target volume (CTV). Two CTVs were defined: CTV1 including prostate and seminal vesicles and CTV2 corresponding to the prostate only.

### 2.2 Workflow

**Figure 1:**
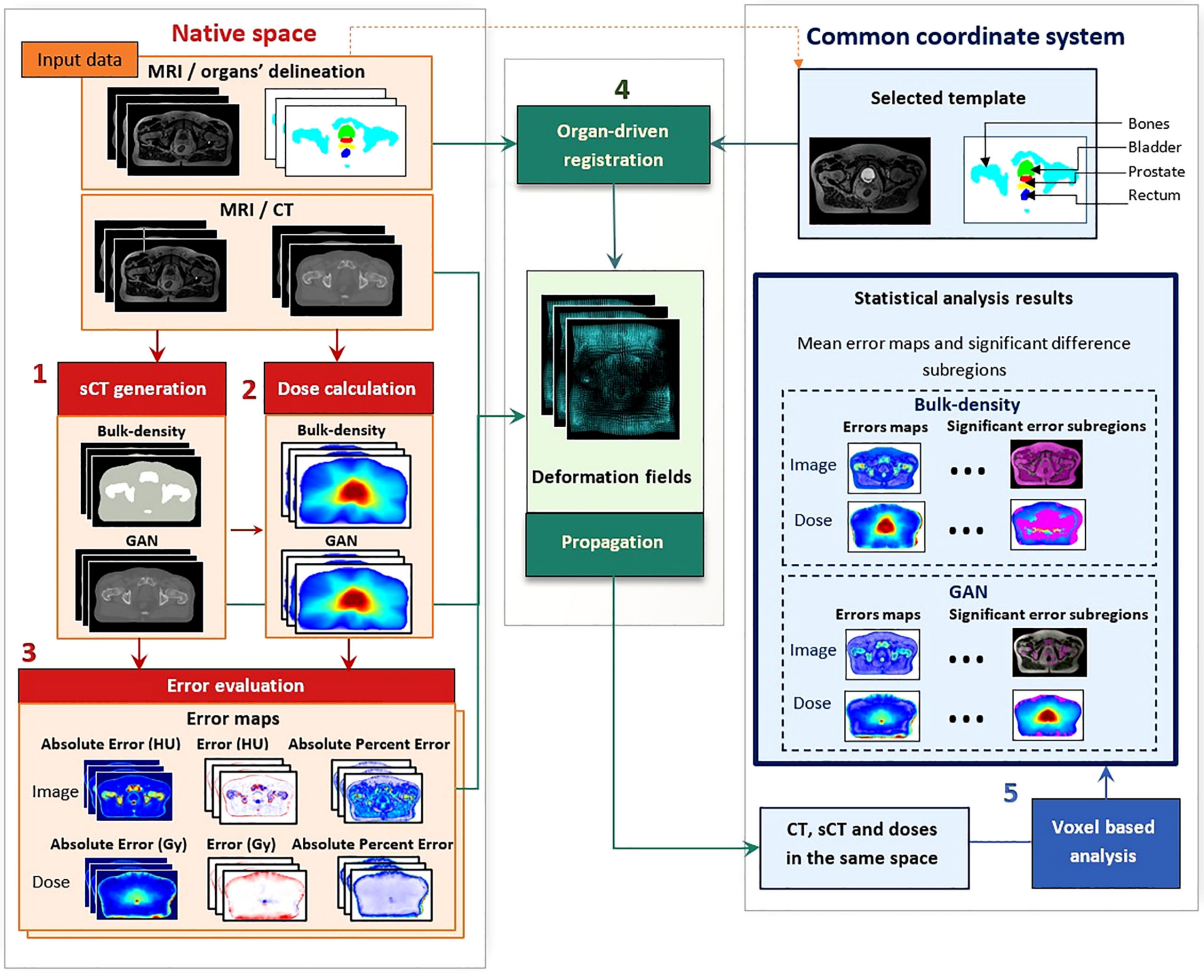
Workflow of voxel-wise population-based analysis. This workflow comprises five steps: (1) synthetic CT (sCT) generation with bulk-density and generative adversarial network (GAN) methods, (2) dose calculation and (3) error evaluation of images and doses in the native space of each patient. This evaluation includes the computation of absolute error, error, and absolute percent error. The non-rigid registration step (4) resulted in deformation fields, allowing for propagation of the whole data to a common coordinate system. Once all data were in the same anatomical space, statistical analysis was performed (5), producing three-dimensional (3D) error maps for each sCT generation method and highlighting significant difference subregions for both image and dose distributions.

The proposed workflow is presented in **Figure 1**. It includes the generation of sCTs using two methods (BDM and GAN) and dose computation. Then, sCTs and dose distributions followed a standard evaluation in the native space. Finally, an accurate customized organ-driven non-rigid algorithm was applied to bring all the data to the same coordinate system, where voxel-wise analysis was performed.

### 2.3 Synthetic CT generation methods

#### 2.3.1 Bulk-density method

BDMs have an application to the quality assurance (QA) of sCT scans (4) and are also employed in this work to demonstrate that the differences between scan quality for different sCT methods can be determined with our workflow. sCTs were obtained by assigning HU values to the patient’s soft tissue, bones, and air segmented from MRI. For bone segmentation, automatic tools from Varian Eclipse were used on CT. This contour was then rigidly aligned to the MRI scan, and contours were manually adjusted by a research radiation therapist (31). The volume of air resulted from thresholds in the inner part of the rectum delineated on MRI. The soft tissue area corresponds to the subtraction of bones and air from the body contour. A water equivalent density (0 HU) was assigned to the soft tissue (3, 32). For bones and air, the densities allocated were 350 and -450 HU, respectively, which are the mean CT values of the cohort in the corresponding segmented regions (28).

#### 2.3.2 Generative adversarial network

The GAN architecture used in this study to generate sCT is fully described in Largent et al. (6). The generator was a U-Net inspired by Han et al. (33), with L2 norm as the loss function:


(1) $$L_{G}(I,\;C)={\left|\left|C-G(I)\right|\right|}_{2}^{2}$$

where *I* corresponds to the MRI intensity, *G*(*I*) to the generated sCT, and *C* to the reference CT.

The discriminator was a PatchGAN, using binary cross-entropy as the loss function:


(2) $$L_{D}(G(I),\;C)=-\sum_{i=1}^{n}C_{i}\log(G{\left(I\right)}_{i})+(1-C_{i})log(1-G{\left(I\right)}_{i})$$

*G*(*I*) is the sCT produced by the generator from the target MRI, *C* is the corresponding reference CT, and *n* is the number of voxels in *C*.

*L_G_* (*I*,*C*) and *L_D_* (*G*(*I*),*C*) were combined to create the adversarial loss.

Axial two-dimensional CT and MRI slices were used to train the model, and threefold cross-validation was applied. The training cohort comprised 26 patient data and a validation cohort size of 13.

### 2.4 Dose calculation in native space

Volumetric modulated arc therapy (VMAT) was planned on reference CT images with the Pinnacle v.9.10 (Philips Healthcare, Cleveland, OH, USA) treatment planning system (TPS) using the collapsed cone convolution algorithm and a dose grid resolution of 3 mm. For all patients, a sequential treatment was delivered with a total dose of 50 Gy to the CTV1 followed by a boost of 28 Gy in the CTV2, both at 2 Gy per fraction. The beam parameters used to compute the dose on the reference CT were used to calculate the dose on the sCT.

### 2.5 Image and dose error evaluation in native space

The accuracy of the sCT generation in HU and in Gy was first assessed in the native space to reduce bias induced by the interpatient non-rigid registration.

Absolute error (AE), error (E), and absolute percent error (APE) were computed by comparing corresponding CT and sCT pairs at a voxel level, producing three-dimensional (3D) error maps for each patient.

The global quality of sCT was evaluated with respect to the patient’s structures (prostate, rectum, and bladder) and whole pelvis by computing the mean absolute error (MAE), mean error (ME), and mean absolute percent error (MAPE) in these regions from the previous maps.


(3*a*) $$AE(i)=|X_{CT}(i)-X_{sCT}(i)|$$

(3*b*) $$MAE=\frac{1}{n}\sum_{i=1}^{n}AE(i)$$

(4*a*) $$E(i)=X_{CT}(i)-X_{sCT}(i)$$

(4*b*) $$ME=\frac{1}{n}\sum_{i=1}^{n}E(i)$$

(5*a*) $$APE(i)=\left|\frac{X_{CT}(i)-X_{sCT}(i)}{X_{CT}(i)}\right|$$

(5*b*) $$MAPE=\frac{1}{n}\sum_{i=1}^{n}APE(i)$$

with *n* being the number of voxels, and *X_CT_* (*i*) and *X_sCT_* (*i*) the intensities of the *i^th^* voxel in, respectively, the reference and the generated image, in HUs for image evaluation or in Gy for dose evaluation.

The closer to zero the AE, E, APE, and so their respective means, the more accurate the prediction.

### 2.6 Organ-driven registration

First, an individual MRI scan from the cohort was selected as a template (exemplar) by considering the median volumes of the bladder, rectum, and prostate. Then, a customized organ-driven registration based upon previously proposed methods (25, 34) was performed with overall optimized alignment across the organs.

Input images for the registration were a combination of the MR images and structural descriptions (SDs) of the delineated organs obtained as follows:

- Euclidean distances to the surface were computed for all structures (35).
- For the rectum, a scalar field was generated by applying the Laplacian equation inside the volume (36). The Laplacian field provided a normalized distance map to the central path of the organ.
- For the prostate, the Laplacian was also computed with respect to its barycenter.

**Figure 2:**
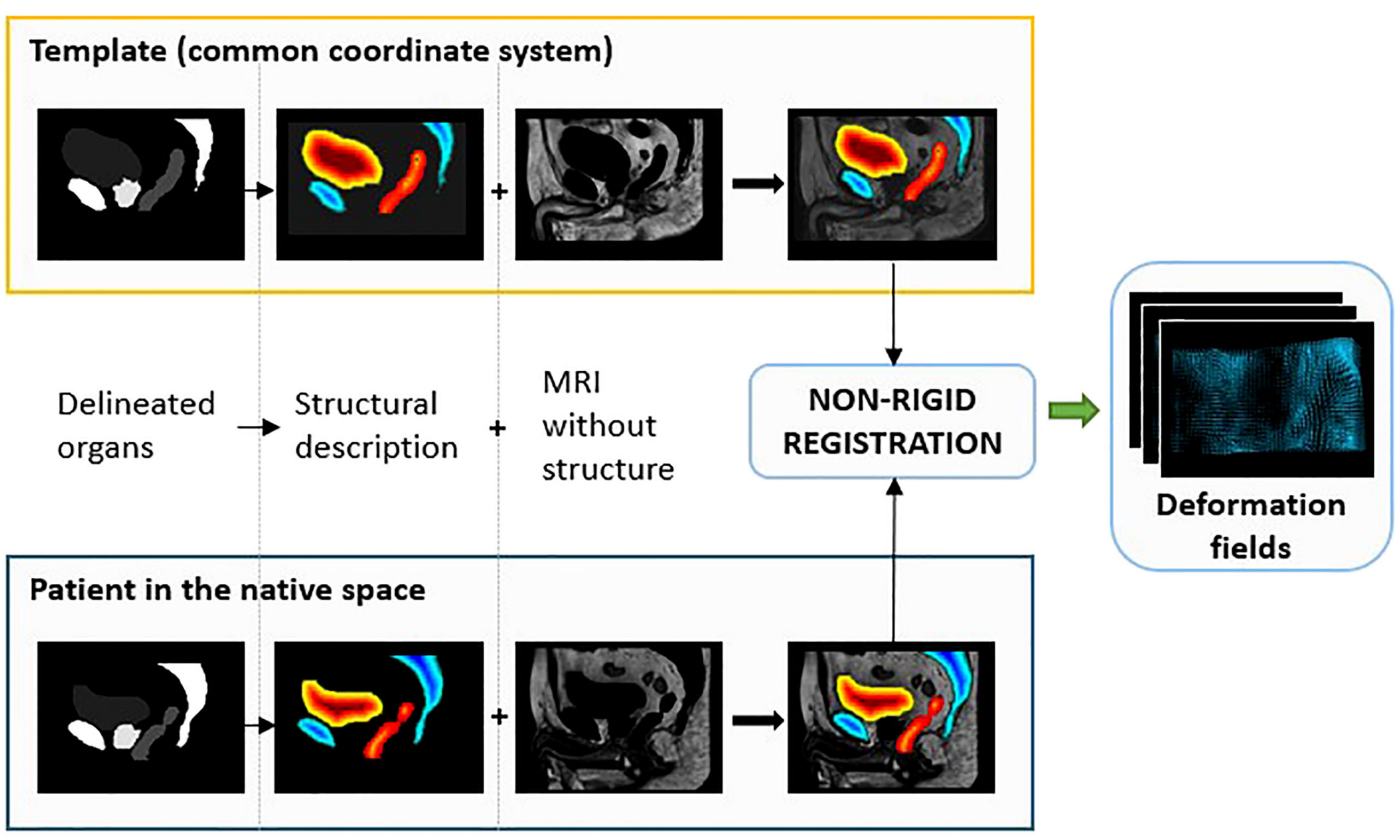
Preprocessing step for the non-rigid registration process. After organ delineation, a structural description was performed by computing the Euclidean distances to the surface and the Laplacian equation. This was finally combined to MR images to obtain the deformation fields used to bring all the data from their native space to the common coordinate system (CCS).

Finally, the scalar fields of all structures were merged into a global structural description of the organs and combined to the MRI (**Figure 2**). Afterward, all the structures were rigidly aligned using the Elastix toolbox (translation). From bones to the bladder, each structure requires a different level of deformation. To handle this high variability, non-rigid registration based on diffeomorphic Demons (37) with four levels of resolution was successively applied to the i) bladder, ii) whole pelvis, iii) prostate, iv) rectum, and v) bones.

The Demons algorithm uses Gaussian regularization, which involves smoothing the deformation field. The sigma of the Gaussian filter was set to 1, and the numbers of iterations for the four levels of resolution were i) 300, 300, 200, and 20 for the bladder contour; ii) 200, 200, 100, and 0 for the whole pelvis; iii) 200, 200, 150, and 5 for the prostate SD; iv) 100, 100, 100, and 5 for the rectum SD; and v) 100, 100, 150, and 50 for the bones SD.

For the bladder, a b-spline transform using the Elastix toolbox was also performed on SD prior to the Demons registration (step i).

Each step resulted in deformation fields: 3D vectors defined at each voxel and providing the appropriate transformation. The resulting 3D deformation fields were combined and applied to delineated structures, reference CTs, sCTs, dose planning, and error maps to propagate all the data from their native spaces to a common coordinate system (CCS).

After the propagation of CT in the CCS, the bones, including the femoral heads, were split between spongy and cortical and separately registered to preserve their inner structure composition. This final transformation was then applied to sCT, dose, and error maps.

For the propagation of CT in the CCS to be meaningful, each CT–MRI patient pair had to be properly coregistered prior to the interpatient registration.

This step-by-step approach can accommodate the high anatomical interindividual variability and facilitates the propagation of delineated structures, including the registered reference CTs, sCTs, dose distributions, and error maps from their native spaces to a CCS.

**Figure 3:**
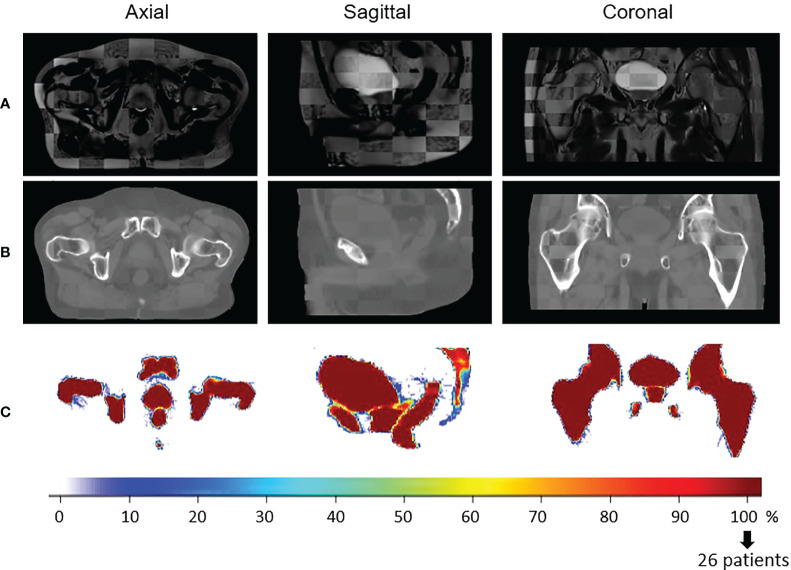
Visual quality control of the interpatient registration. Checkerboard comparison of **(A)** the template MRI with the mean of all the population MRIs registered in the Common coordinate system (CCS) and **(B)** the template CT with the mean population CTs in the CCS. Probability maps are presented in **(C)**. It is the result of the overlapping of all the delineated structures in the same space to estimate the precision of the registration. In blue, few structures are overlaid (poor quality of registration). In red, all the patient structures correspond to the same anatomical location (100%, perfect registration).

As a visual indicator of the performance of this process, a checkerboard of the template MRI with the mean population MRI in the CCS and a checkerboard of the template CT with the mean population CT in the CCS are presented in **Figure 3**. The probability maps, also in **Figure 3**, allow the visualization of the discrepancies between the delineated organ contours following registration.

**Table 2** summarizes the volumes of the delineated organs prior to and after the registration process.

The Dice similarity coefficient (DSC) between the template structures, Vt_MRI, and the corresponding deformed delineated organ, VMRI, was also used for validation.


(6) $$DSC=\frac{2(V_{t\_MRI}\cap V_{MRI})}{V_{t\_MRI}+V_{MRI}}$$

For the voxel-based population analysis to be meaningful, only accurately registered data were included (DSC > 0.85 for all the segmented organs). The 26 cases passed this criterion.

### 2.7 Voxel-wise analysis in common coordinate system

#### 2.7.1 Image and dose mean error map computation

Once all data were in the CCS, voxel-wise MAE (vMAE), ME (vME), and MAPE (vMAPE) maps for images and dose distributions were obtained by averaging the voxel error data across the cohort. *v* represents that these data are now voxel-specific and hence spatial, i.e., they are not averaged across a particular patient’s voxels; they are found by considering all the patient cohort values for a particular voxel *i*.

Therefore, in the CCS, errors are defined as follows:


(7) $$vMAE(i)=\frac{1}{p}\sum_{j=1}^{p}\left|X_{CT}(i,j)-X_{sCT}(i,j\right)|$$

(8) $$vME(i)=\frac{1}{p}\sum_{j=1}^{p}X_{CT}(i,j)-X_{sCT}(i,j)$$

(9) $$vMAPE(i)=\frac{1}{p}\sum_{j=1}^{p}\left|\frac{X_{CT}(i,j)-X_{sCT}(i,j)}{X_{CT}(i,j)}\right|$$

*vMAE*(*i*) is the mean absolute error, *vME*(*i*) the mean error, and *vMAPE*(*i*) the MAPE for a voxel *i*. *X_CT_* (*i, j*) and *X_sCT_* (*i, j*) represent the values, in HUs for the image assessment or in Gy for the dose assessment, of the reference CT and the sCT, respectively, for the *i^th^* voxel of the *j^th^* image of the population, and *p* is the total number of patients in the population.

The template scan body contour was applied to these images to focus on the region of interest and discard slight body contour variation due to registration. Then, the relative standard deviation of the absolute error (RSD_AE_), also known as the coefficient of variation, was used for the evaluation of the dispersion of the prediction error at a voxel-wise scale.


(10) $$RSD_{AE}(i)=\frac{\sqrt{\sum_{j=0}^{p}{\left(AE(i,j)-vMAE(i)\right)}^{2}}}{vMAE(i)}$$

with *AE*(*i, j*) = |*X_CT_* (*i, j*) – *X_sCT_* (*i, j*)|

**Figure 4:**
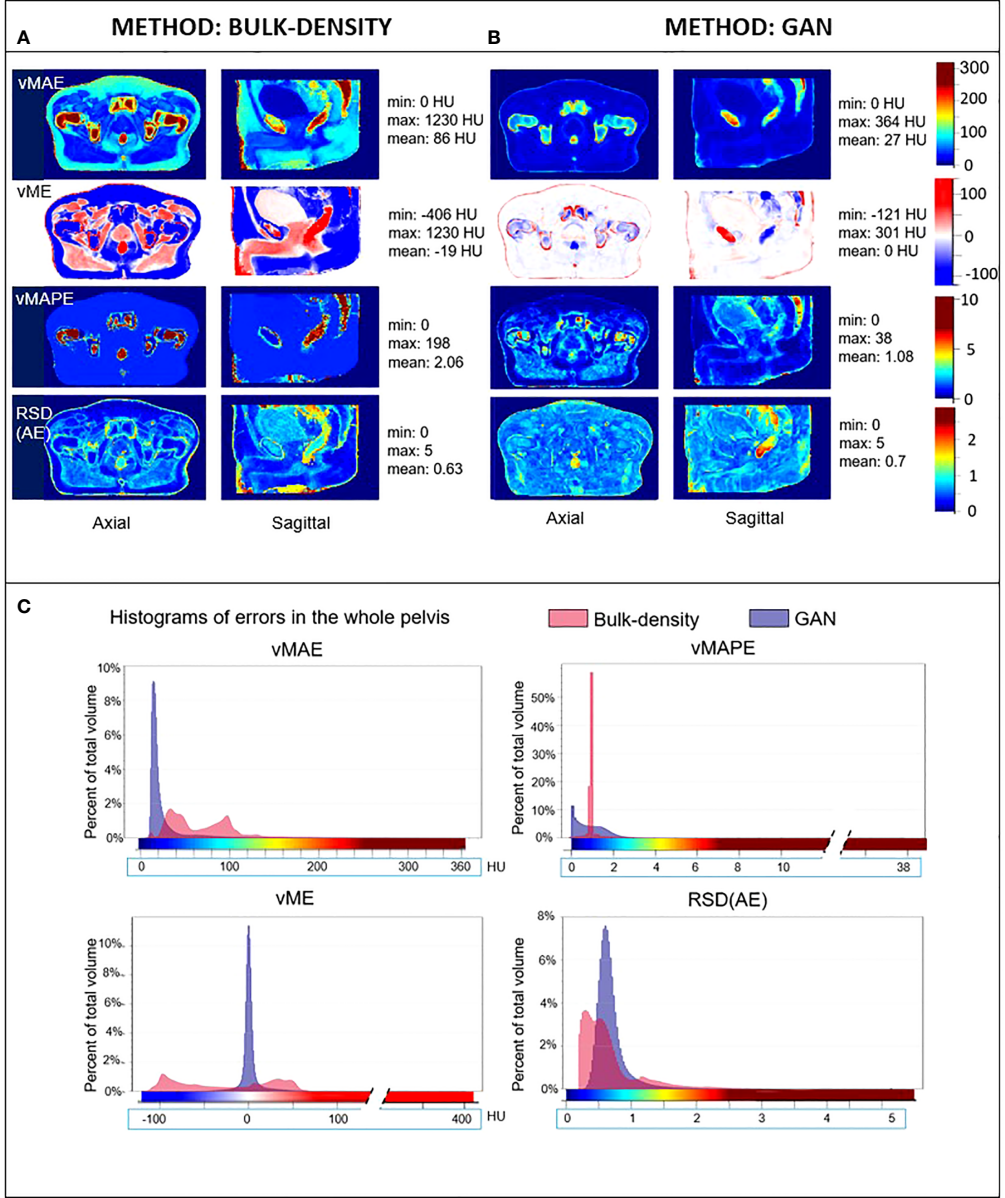
HU error maps in the common coordinate system. Axial and sagittal views of voxel-wise mean absolute error (vMAE), voxel-wise mean error (vME), and voxel-wise mean absolute percent error (vMAPE) maps in the same anatomical space and the corresponding histograms **(C)** for sCT generated with the **(A)** bulk-density and **(B)** GAN methods. The relative standard deviation of the absolute error [RSD(AE)] is also illustrated. Color scales of error maps were associated to histograms.

**Figure 5:**
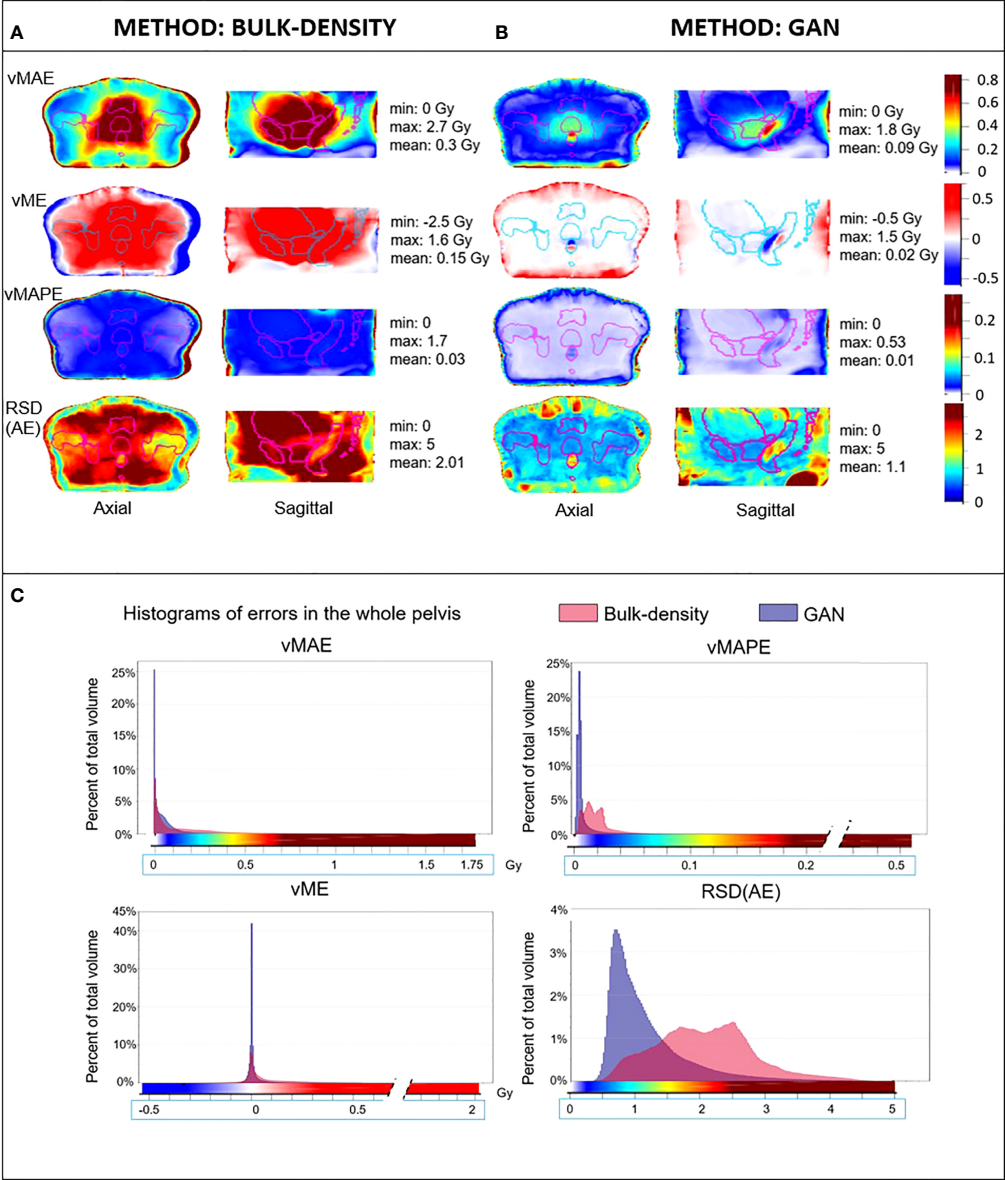
Mean dose error maps in the Common coordinate system (CCS). Axial and sagittal views of vMAE, vME, and vMAPE maps in the same anatomical space and the corresponding histograms **(C)** for dose computed from sCT generated with **(A)** bulk-density and **(B)** GAN methods. The RSD(AE) is also illustrated. Contours of the delineated organs of the template were overlaid on each image, and the color scales of error maps were associated to histograms.

Therefore, for each voxel *i*, the lower the RSD_AE_, the higher the probability to have an absolute error close to the *vMAE*(*i*) value. **Figures 4**, **5** illustrate the results, respectively, for image and dose assessment.

#### 2.7.2 Permutation test

**Figure 6:**
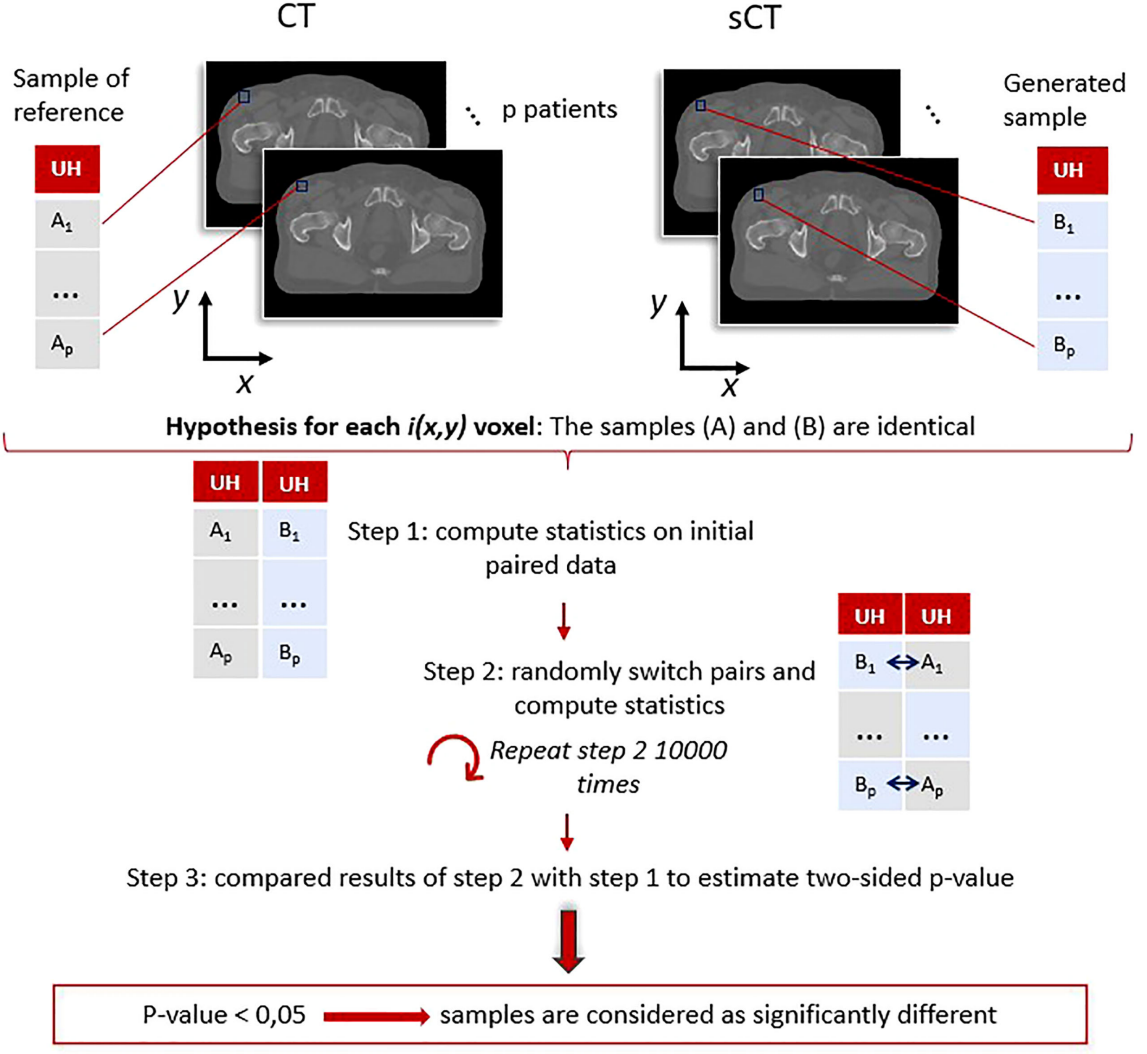
Paired permutation test general workflow: example for the image evaluation using Hounsfield units. For each voxel, coordinates (x,y) correspond to paired data (A_1_, B_1_), …, (Ap, Bp). These pairs were used to determine if the generated (B) and reference (A) samples were identical or not following the procedure proposed by Konietschke et al. (38). A p-value (x,y) is obtained for each voxel, highlighting the regions where the differences are significant. The same process was applied on dose distributions.

To complete this study, voxel-wise paired permutation tests proposed by Konietschke et al. (38) were performed for each method with the R software package for non-parametric multiple comparisons (39). This statistical approach is an adaptation of Student’s t-test for non-parametric paired data and includes permutation tests. The hypothesis in this study was that the intensity in HUs, or the dose in Gy, of the generated sCT scans was identical to the value of the reference scans (**Figure 6**).

Two paired lists of values were determined for each voxel and compared.

Multiple comparisons may lead to type I errors, namely the false-positive rate. Therefore, to limit these errors, 10,000 random permutations were utilized to estimate the p-value.

The procedure to estimate the p-value followed these steps:

- The computation of the statistics (38) on the initial data: *U* = (*U*_1_, ···,*U_p_*), with *U*_1_ = (*X_CT_* (1),*X_sCT_* (1)) the paired values for patient 1, and p the total number of patients in the population.
- The computation of the statistics on randomly permuted data defined as *U_perm_* = (*U_perm_*_1_, , *U_permp_*), with *U_perm_*_1_ = {((*X_CT_* (1), *X_sCT_* (1)), ((*X_sCT_* (1), *X_CT_* (1))} the two possible paired values for patient 1. This step was repeated 10,000 times.
- The comparison of the results obtained with the swapped data *U_perm_* and the one obtained in the first step to estimate the p-value (38).

**Figure 7:**
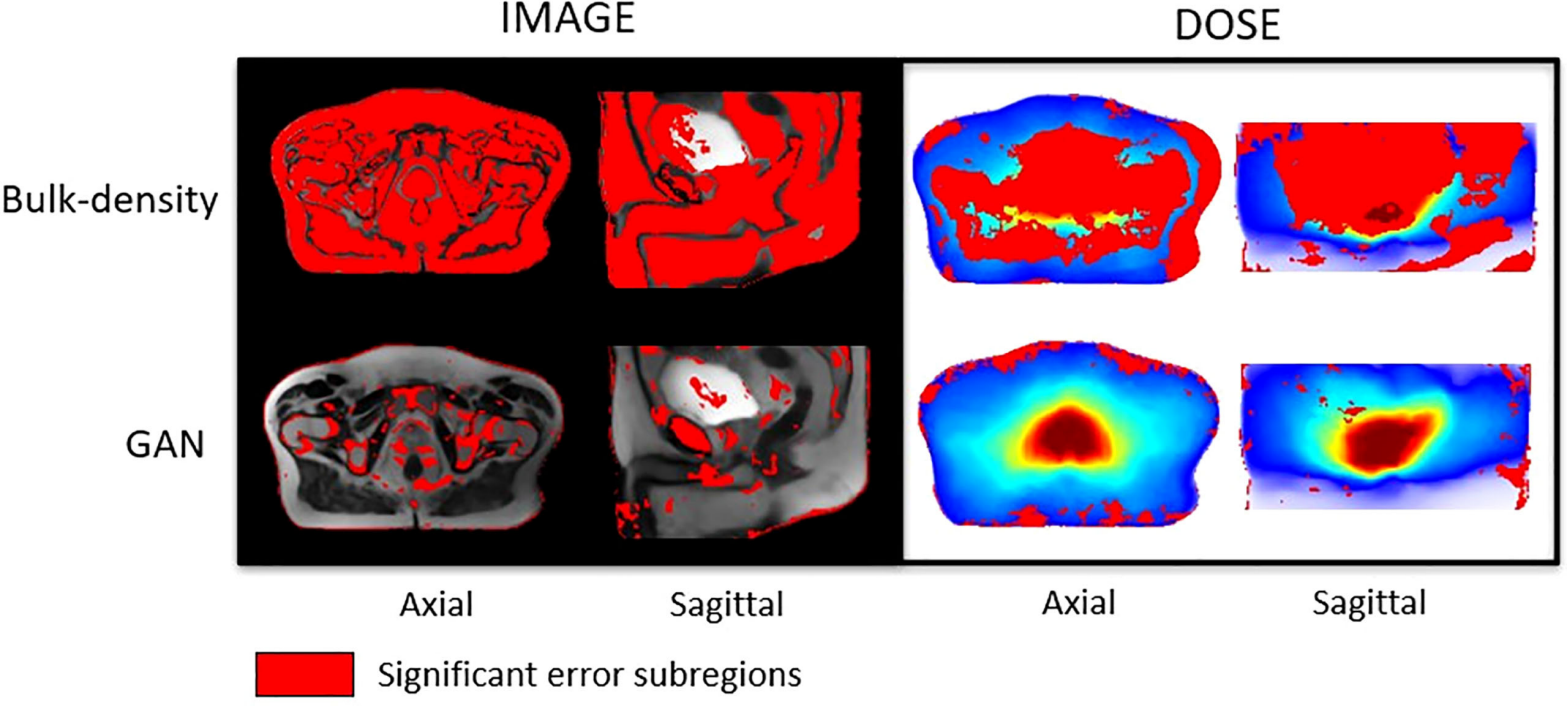
Studentized paired permutation test results. Significant error subregions brought out by Konietschke’s paired permutation test, in red, overlaid on mean MR images in the CCS for HU values (left) and overlaid on the mean dose plans in the CCS for Gy values (right). This statistical test produced p-value maps. Differences of intensities (HU) on one hand, and dose (Gy) on the other hand, were considered as significant for p-value< 0.05.

This test resulted in 3D maps, where a voxel *i* corresponds to the probability that the initial hypothesis was true for the *i^th^* voxel of the generated sCTs. The regions of significant differences (*p-value*< 0.05) between CTs and sCTs on one hand and between dose plans calculated on CTs and sCTs on the other were generated. These volumes, referred to as error subregions (ESRs), are illustrated in **Figure 7**.

### 2.8 Mean absolute dose error—volume histogram

This cumulative histogram is a quantitative tool, allowing for the assessment of absolute error in the dose calculations on the sCT and CT scans with respect to the volumes of tissue. It was built in the same way as DVHs and computed from the vMAE map in the CCS. The regions of interest for this evaluation were the bladder, rectum, prostate, and pelvis. To focus on the region of the dose distribution, the pelvic region was cropped to within 2 cm above and 2 cm below the rectum, according to the superior-to-inferior axis.

Two criteria for evaluation were selected: V_0.5Gy_ and V_1Gy_, which correspond, respectively, to the total volume with an absolute error greater than or equal to 0.5 and 1 Gy.

### 2.9 Dosimetric endpoints

#### 2.9.1 Gamma analysis

Dose plans were propagated to the CCS and combined, resulting in the mean reference CT dose and mean dose for each sCT generation method. Thus, a spatial dose evaluation was conducted comparing mean dose distributions with a 3D gamma analysis (local, 1%/1 mm, dose threshold 10%) using VeriSoft software. The gamma pass rate, corresponding to the percentage of voxels with gamma inferior to 1, and mean gamma were reported, additionally to gamma maps in the axial plan.

#### 2.9.2 DVH criteria

The absolute differences between dosimetric values calculated on the reference CT propagated in the CCS and those calculated using sCT generated from the BDM and the GAN were determined. The contours used were the bladder, rectum, and prostate of the template in the CCS.

**Table 4** presents the average differences of the mean dose, D2%, D50%, and D95% for each method, with Dx% representing the dose in x% of the volume of interest.

## 3 Results

### 3.1 Image and dose error evaluation in native space

**Table 1** depicts the results of the evaluation in the native space for both bulk-density and GAN methods. The BDM presented higher MAE, MAPE, and ME than the deep-learning-based approach. The worst MAE scores for both methods were in the bone regions (244.4 HU for the BDM and 124.3 HU for the GAN). This structure also had a higher mean CT number and standard deviation (342 HU ± 317 HU).

**Table 1:** Error evaluation performed in the native space for sCT generation methods.

			IMAGE (HU)			DOSE (Gy)		
			GAN	BULK-DENSITY	Mean CT number	GAN	BULK-DENSITY	Mean dose
		MAE	**33.9 ± 7.6**	96.4 ± 16.5		**0.06 ± 0.02**	0.2 ± 0.36	
Global	PELVIS	MAPE	**1.3 ± 0.6**	2.3 ± 0.8	18 ± 184	**0.1 ± 0.03**	0.12 ± 0.04	8.9 ± 13.4
		ME	**3.4 ± 15.6**	-10.4 ± 24.3		**0.11 ± 0.05**	0.19 ± 0.27	
		MAE	**124.3 ± 22.4**	244.4 ± 29.8		**0.06 ± 0.03**	0.24 ± 0.46	
	BONES	MAPE	**1.3 ± 0.8**	3.9 ± 1.8	342 ± 317	**0.04 ± 0.02**	0.06 ± 0.03	14.6 ± 15.1
		ME	23.9 ± 45.7	**20.4 ± 62.3**		**0.03 ± 0.08**	0.24 ± 0.47	
		MAE	18.2 ± 4.9	**17.1 ± 5.8**		**0.11 ± 0.1**	0.72 ± 1.88	
	BLADDER	MAPE	2.2 ± 1.2	**1.1 ± 0.1**	4 ± 19	**0.01 ± 0.01**	0.02 ± 0.05	25.8 ± 22.7
Organ-		ME	4.9 ± 12.0	**4.9 ± 12.9**		**-0.02 ± 0.15**	0.69 ± 1.89	
wise		MAE	**67.1 ± 66.6**	140.9 ± 71.8		**0.23 ± 0.23**	0.79 ± 1.62	
	RECTUM	MAPE	**2.1 ± 1.2**	6.8 ± 6.2	-13 ± 135	**0.01 ± 0.0**	0.02 ± 0.05	36.7 ± 19.2
		ME	**-16.3 ± 77.6**	98.2 ± 82.9		**-0.04 ± 0.18**	0.58 ± 1.68	
		MAE	**17.6 ± 3.8**	34.2 ± 8.5		**0.34 ± 0.2**	1.46 ± 3.54	
	PROSTATE	MAPE	1.2 ± 1.0	**1.0 ± 0.0**	29 ± 24	**0.0 ± 0.0**	0.02 ± 0.05	78.7 ± 0.8
		ME	**3.7 ± 11.3**	30.7 ± 11.6		**-0.04 ± 0.38**	1.3 ± 3.61	

Global scores for the whole pelvis and per organ are presented. Mean absolute error (MAE), mean absolute percentage error (MAPE), and mean error (ME) were computed between reference CT and sCT (image results in HU) and between dose distribution calculated from these images (dose results in Gy). Reference CT number and mean dose in each anatomical region are also indicated.

Bold values highlight best metrics scores.

Regarding dose calculation, MAE reached 1.46 Gy, equivalent to 1.85% of the expected dose, in the prostate for the BDM and 0.34 Gy for the GAN. For each method, MAPE was similar for the prostate, rectum, and bladder (approximately 0.02 for the BDM and 0.01 for the GAN) and superior in bones (0.06 and 0.04). The standard deviation for all error types and all delineated organs was larger for the BDM compared to the GAN.

### 3.2 Registration

The customized non-rigid registration process accurately brought the 26 patients of the cohort in the same anatomical space, as shown by the average Dice score of 0.98 ± 0.01 for the body contour, 0.93 ± 0.01 for the bones, 0.96 ± 0.01 for the bladder, 0.91 ± 0.02 for the rectum, and 0.91 ± 0.02 for the prostate. The mean volume, in cubic centimeters, of each delineated structure ended close to the volume of the template’s organs in the CCS (**Table 2**) confirming the efficiency of the method.

**Table 2:** Volume of delineated structure in cm^3^ prior and after the non-rigid registration.

	VOLUME IN NATIVE SPACE (cm^3^)				REGISTERED VOLUME (cm^3^)				TEMPLATE IN CCS (cm^3^)
	mean	std	min	max	mean	std	min	max	
**BODY**	14362	2092	10608	18300	15392	261	14363	15812	15374
**BLADDER**	274	142	113	633	243	3	237	251	246
**BONES**	1259	205	908	1817	1082	36	1031	1183	1076
**PROSTATE**	40	19	16	82	33	1	31	37	34
**RECTUM**	66	29	25	133	36	1	34	37	36

These data are presented regarding the volume of the template in the common coordinate system (CCS).

The accuracy of the registration inside the body is also illustrated visually in **Figure 3**.

### 3.3 Voxel-based error maps

#### 3.3.1 Image assessment

**Figure 4** depicts the vMAE, vME, and vMAPE error maps computed in the CCS for both the BDM and the GAN method. The RSD_AE_ map, representing the dispersion of the absolute error distribution at each voxel considering the overall cohort, is also included. It illustrates the voxel-wise quality assessment of sCT generated for each method. The histograms of these 3D error maps are presented in this figure, which allows the comparison of the accuracy of both methods. Differences in intensity up to 250 HU in the rectum and more than 500 HU in cortical bones were found for the BDM. An underestimation (in red, **Figure 4**) of more than 200 HU in the cortical bones and approximately 140 HU in the rectum were observed in the sCT generated from the BDM, as well as an overestimation (in blue, negative values) of 200 HU in spongy bones. For the GAN, the highest vMAE was found in bones (approximately 100 HU and up to 220 HU in denser regions). The vMAE reached 200 HU in a small specific region within the rectum, close to the prostate and seminal vesicles. According to the vME map, the GAN approach led to an overestimation (in blue, **Figure 4**) in the previously described location in the rectum, with a score equal to -85 HU, and in spongy bones (-40 HU). There was an underestimation of 110 HU in cortical bones (in red, **Figure 4**). The errors highlighted with the vMAPE were in spongy bones and in the rectum for both methods, also in the contour of the bladder for the GAN. The vMAPE histogram for the BDM has a narrow distribution around 1 in soft tissue, as computing the MAPE in this area, where the sCT value is equal to 0 HU, results in dividing the reference CT value by itself. Although the RSD_AE_ was more than 1.5 and 2, respectively, for the BDM and the GAN in the rectum, the highest values were not at the same location.

**Figure 7** presents significant ESRs, in red, overlaid on the mean MR images in the CCS and on the mean dose distribution. Most of the HU values predicted with the BDM were significantly different from the reference CT HU values, except in an important part of the bladder and tissue interfaces. According to the studentized permutation test result, ESRs were preferentially located in cortical bones, skin, a part of the prostate, and regions scattered around the bladder and the rectum for the sCT obtained with the DLM.

#### 3.3.2 Dose assessment

**Figure 5** illustrates the dose differences for the whole population data. As for the image assessment, the resulting maps allowed to evaluate and compare locally resulting in the dose calculation of both sCT generation methods. For the BDM, vMAE in the organs at risk increased up to 1.7 Gy, just near the prostate. The most predominant absolute errors for the GAN appeared in the rectum with differences up to 0.75 Gy and the first centimeter of the body contour. In the prostate, the vMAE was approximately 0.3 Gy. The vME reached 0.4 Gy on the body contour for the DLM. The vMAPE confirmed the error on the body contour but not in the rectum for both approaches. RSDAE highlighted the same area in the rectum than vMAE and vME maps (RSDAE > 1.5). The higher the delivered dose, the higher the error observed, with an underestimation of the dose distribution of 1.3 Gy in the prostate for the BDM. As for image analysis, dose error map histograms appeared wider than for the GAN (**Figure 5**).

According to **Figure 7**, a major part of the dose plans computed from the BDM was considered as significantly different from the ground truth. For those calculated from sCT generated with the GAN, ESRs were localized surrounding the body, mainly on the skin and until 3 cm inside the body.

### 3.4 Mean absolute dose error per volume

**Figure 8:**
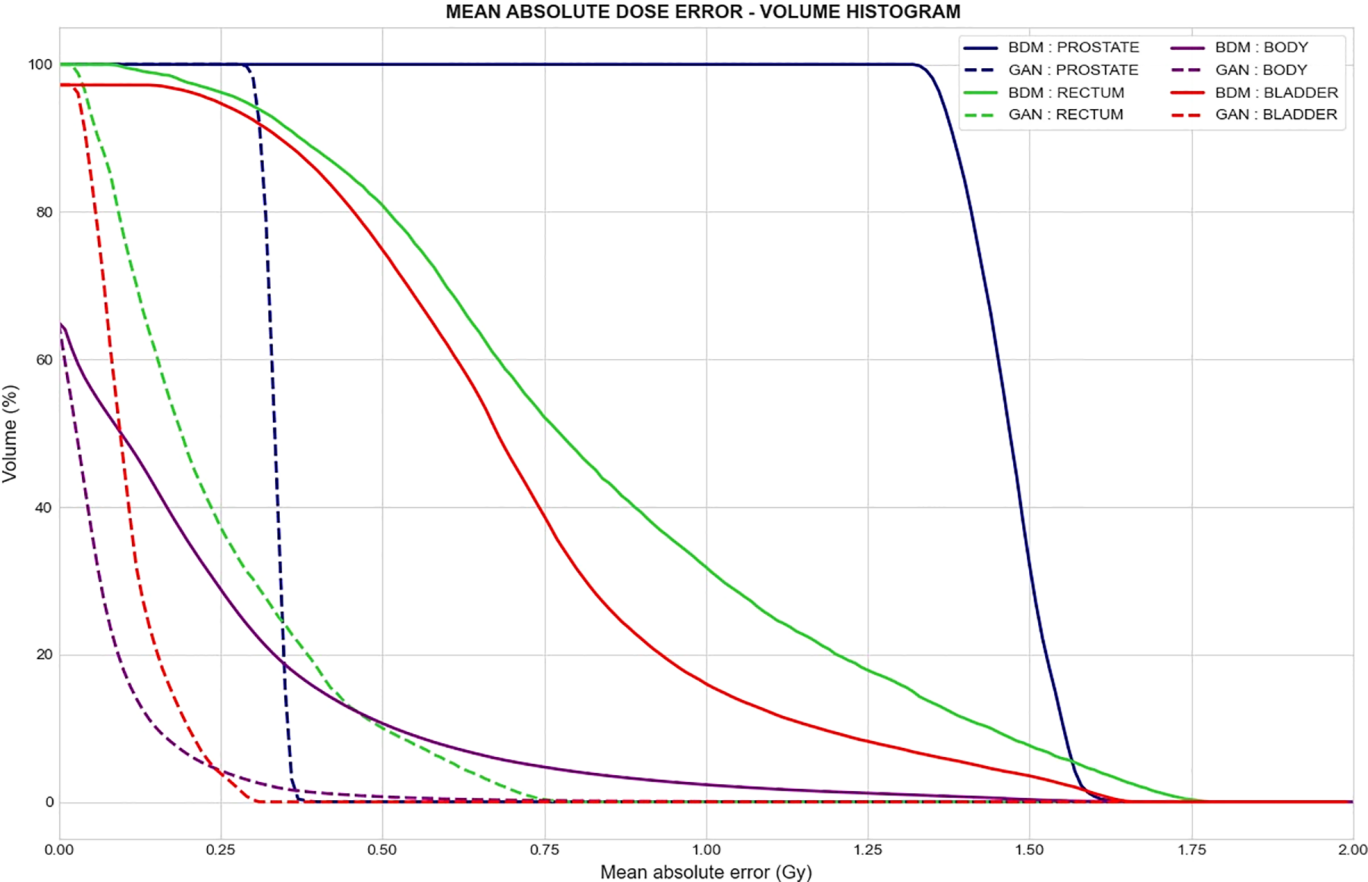
Mean absolute dose error–volume histogram. Mean absolute difference between dose computed from the reference CT and dose computed from the synthetic CT generated with the bulk-density method (continuous line) and GAN (dotted line) for a specific volume of delineated structures. Each color represents a tissue volume.

**Figure 8** presents the comparison of the two sCT methods by showing the absolute dose difference (Gy) per percentage of tissue volume. This metric reveals a larger error for the BDM than the GAN, regardless of the organ considered. No volume reached 1 Gy of dose difference for the GAN sCT (**Table 3**).

**Table 3:** Percent of tissue volume with a mean absolute error (MAE) reaching 0.5 Gy (V_0.5 Gy_) and 1 Gy (V_1 Gy_) for both sCT generation methods.

	BULK-DENSITY				GAN			
	PELVIS	BLADDER	RECTUM	PROSTATE	PELVIS	BLADDER	RECTUM	PROSTATE
V_0.5 Gy_	16.58%	77.03%	80.93%	100%	1.10%	0%	10.03%	0%
V_1 Gy_	3.63%	16.48%	31.85%	100%	0.08%	0%	0%	0%

The mean of voxel values of the vMAE map in the CCS was computed in the whole pelvis and in the template’s structures (bladder, rectum, and prostate).

### 3.5 Dosimetric endpoints

**Figure 9:**
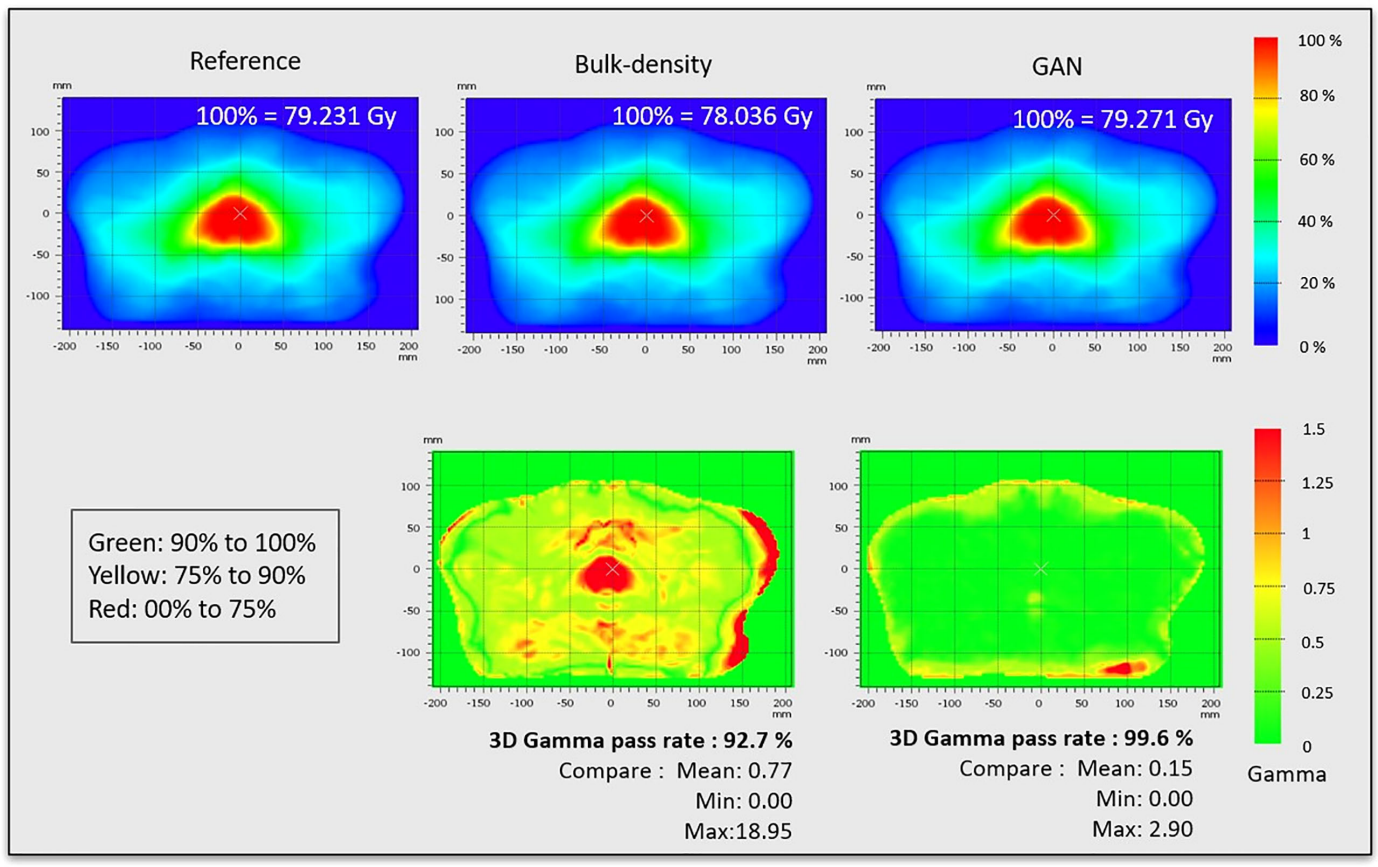
Dose distributions and gamma maps. Dose distributions were propagated to the CCS and combined, resulting in mean reference CT dose, mean dose for sCT generated from bulk density, and mean dose for sCT generated from GAN method. These dose distributions were used to calculate the gamma pass rate (criteria: 3D, local, 1%/1 mm, low dose threshold = 10%).

The results of 3D gamma analysis (criteria: local, 1%/1 mm, low dose threshold = 10%) performed on the mean dose volume in the CCS are presented in **Figure 9**. This allows for a local comparison of the gamma maps of each sCT generation method.

In **Table 4**, dosimetric criteria assessment shows an absolute difference superior to 1 Gy in the prostate for the BDM, while the GAN results are around 0.33 Gy in this location.

**Table 4:** Absolute difference of dosimetric criteria computed for both bulk-density and GAN methods using the template contours in the Common coordinate system (CCS).

	BULK-DENSITY			GAN		
	BLADDER	RECTUM	PROSTATE	BLADDER	RECTUM	PROSTATE
**Mean dose absolute difference (Gy) ± std**	0.71 ± 1.90	0.69 ± 1.85	1.45 ± 3.62	0.10 ± 0.10	0.16 ± 0.12	0.33 ± 0.21
**D2% absolute difference (Gy) ± std**	1.59 ± 3.66	1.44 ± 3.28	1.41 ± 3.58	0.27 ± 0.19	0.50 ± 0.62	0.33 ± 0.22
**D50% absolute difference (Gy) ± std**	0.67 ± 1.88	0.66 ± 1.68	1.43 ± 3.63	0.09 ± 0.09	0.18 ± 0.20	0.33 ± 0.21
**D95% absolute difference (Gy) ± std**	0.24 ± 0.83	0.22 ± 0.56	1.49 ± 3.63	0.03 ± 0.05	0.04 ± 0.04	0.32 ± 0.22

Absolute difference of the dose means, D2%, D50%, and D95% computed between the reference CT and the synthetic CTs in the rectum, bladder, and prostate. Dx% represents the dose in x% of the volume of interest.

## 4 Discussion

This study proposed a methodology based on voxel-wise population analysis to assess the local errors in sCT generation approaches and their impact on the dose distribution. It also allows the comparison of the performance of several sCT generation methods. The full evaluation process was applied on two sCT generation methods, allowing for the examination of heterogeneity of errors in not only HU but also 3D dose distributions across the pelvis.

The presented methodology relies on the accuracy of the interindividual non-rigid registration step, as for all voxel-based approaches (40). Registration methods have been developed in morphometry studies (41–43). Previous studies in the pelvic area included the structural descriptions of the bladder and prostate only (25) or rectum only (34) or were combined to CT (44). The voxel-wise statistical analysis performed here includes a novel integration of bones, with a step dedicated to the preservation of their inner structure. The combination of these structural descriptions with MR images is also original in this context and achieved a precise registration of the whole pelvis as it offers superior contrast in soft tissue. With the Demons algorithm for deformable registration, the amount of deformation is limited by the deformation field smoothing at each iteration, which helps avoid large and unnatural displacement. The algorithm is quite robust to breaking down; however, this is possible if the anatomy or modality is very different, particularly if the rigid registration step has failed prior to the Demons algorithm.

The same pelvic MRI data used in this study had been successfully evaluated in previous work that has relied on the same registration method (31, 45). While the reported DSCs highlight the structural similarities, these are also robust indicators for when the analysis would break down. The major displacement of the organs leading to non-realistic deformation within the body during the registration will impact the DSC of the contours and can provide a good QA step to ensure that the registration has not failed. The mean DSC of 0.98 for the body contours indicates that the registration on this dataset appears to be accurate.

This method permits to map organs, images, and doses in a single coordinate system. Comparison by voxel is thus anatomically meaningful for both images and doses.

vMAE, vME, vMAPE, and RSD_AE_ 3D maps were produced, showing the distribution of mean error across the pelvis for a whole population. The error map histograms are a quantitative tool to compare the chosen methods. As vMAE map values appear to be correlated to the reference intensity (the most important errors are in cortical bones, where the mean HU value is the highest), the relative difference, vMAPE, was also computed as a measure of prediction accuracy. The purpose of vME maps is to determine if the prediction tends to be systematically superior or inferior to the reference, and the RSD_AE_, also known as the coefficient of variation, can be interpreted as the uncertainty maps of each method (46). RSD_AE_ gives an insight into the regions where HU prediction is trustworthy or not. Therefore, each 3D map computed in this study illustrated complementary information on errors produced in both sCT and dose distributions.

To define if the errors were significant across the anatomy in the CCS, a voxel-wise statistical test was applied on images and on dose distributions. The permutation test proposed by Konietschke et al. (38) was used to cope with the multiple comparison problems and is appropriate for paired and non-parametric data. Other permutation tests, such as Chen’s (47) used in Chourak et al. (27), do not appear suitable in our approach as they do not compare each CT to its corresponding sCT.

The two evaluated methods were the BDM and the DLM using the GAN. The BDM is a historical approach for MRI-only radiation planning and was first integrated in a commercialized device (MRCAT, Philips (48)). The BDM also has an application to the QA of sCT scans (4). This approach is simple and does not involve registration, but it lacks accuracy as it does not take tissue heterogeneity into account. The BDM presented in this paper was chosen as an illustration of the proposed methodology, but it has been shown that more accurate methods exist (3, 48–50).

Although several sCT generation methods have been proposed in the literature, recent studies head toward deep-learning strategies (12, 51) DLMs such as the GAN trained with paired data rely on intrapatient registration precision (52). The multimodal registration of the input data and training is time consuming, but generated sCTs are, in general, more accurate (6, 20).

According to the RSD_AE_ map, the GAN was more consistent in HU prediction and resulted in more reliable dose planning. For both methods, important MAEs and MEs arose in the rectum, near the prostate. This area corresponded to a high RSD_AE_ regarding other structures and a high MAPE, expressing the lack of accuracy of both methods in this location. Furthermore, the error did not stand out as significant with the studentized permutation test for the GAN. This wide error might be due to the change in patients’ anatomy between CT and MRI acquisition but is not necessarily related to an incorrect prediction of the HU. Another possibility is that the change in patients’ anatomy disrupted the training phase for the GAN.

The BDM statistically lacked accuracy for HU prediction and dose calculation. For the GAN HU values, significant differences were observed in cortical bones, especially in the femoral heads, but no significant consequences appeared in the dose distribution.

Although HU prediction accuracy is important, sCT generation needs to be reliable for dose planning. Dosimetric assessment is thus crucial and is usually based on DVH, which is an organ-based metric, and gamma analysis. The gamma was computed in the CCS, allowing for the extraction of local values across the population. The location of dose discrepancies is clearly visible, with gamma superior to 1 in the prostate for the BDM (**Figure 9**). Gamma results allow a spatial dose analysis of the sCT generation method for the chosen criteria (1%/1 mm in this study).

Recent studies in sCT generation involve deep learning for different anatomical locations. Nevertheless, artificial intelligence (AI) is not yet fully trusted for clinical use, and key points to assess AI solutions in radiology are raised (53). Critical questions for performance and validation are related to robustness to input variability, training data, and potential sources of bias identified by developers. As the GAN was trained with paired CT and MRI, the multimodal registration accuracy directly impacts the quality of sCT (52). In addition, uncertainties inherent to deep-learning models (54) also generate misprediction.

These uncertainties may produce errors in sCT HU values and so may impact dose computation.

The population-based strategy presented in this paper offers the possibility to define at a voxel level the capability of a method to be accurate across a cohort of patients, having variable tissue density and anatomy, in HU and on the resulting dose distribution. It gives an insight on the reliability of sCT generation, where, usually, the assessment is limited to global or organ-wise assessment (1, 55, 56).

A limitation of the registration process might be the accuracy of the contours. Interobserver delineation for the bladder, prostate, and rectum on a similar dataset appeared to be close in a previous study (31). However, the experts may have been more experienced than the physicians who segmented the data for this project.

Nevertheless, the relations between HU errors and their impact on dose computations are yet to be investigated. *In silico* models with simulated HU errors in specific tissue followed by dose computation could help to determine the acceptable level of error in sCT that will not affect the dose.

Overall, voxel-wise analysis brought out significant differences that did not show up with the global scores and allowed the assessment of both HU prediction and dose distribution. This process identified locations where the sCTs were more prone to errors. This will provide a way forward for translation to a clinical radiotherapy practice. However, the analysis accuracy highly depends on the quality of the interpatient registration. As misregistration can remain, dissociating registration errors to those inherent to the generation methods is an issue of interest and is yet to be fully explored.

Even if the sCT generation method appeared to be accurate, there is no guarantee that each new sCT will be reliable for dose calculation, especially for a patient anatomically different from the training cohort or if the MR image presenting artifacts is acquired with a different sequence or device.

The implemented voxel-based analysis workflow depends on interpatient registration accuracy: a mismatch between structures will lead to biased results. Moreover, the statistical test presented in this paper is time consuming, as simulation studies show that at least 10,000 random permutations are needed for each voxel for an adequate p-value estimation (38). Furthermore, type I errors may remain in the ESR.

This methodology is a tool for assessing and comparing sCT generation methods and illustrating inhomogeneities. However, more studies are required to go further in a QA process. Part of our future work is to investigate the ability to assess a single sCT, without reference, before its use for dose calculation.

This study focused on the male pelvic area considering prostate cancer irradiation; however, the methodology can be applied to any other anatomical location provided that accurate registration is achieved.

## 5 Conclusion

The proposed voxel-wise population-based workflow resulted in 3D error maps for sCT generation from MRI. This methodology relies on a robust organ-driven non-rigid registration that brings all the patients to the same anatomical space. The assessment of HU and dose distributions calculated from sCT accuracy followed a multiscale strategy, whereby errors were computed for the whole pelvis, followed by the organs and finally at a voxel level, allowing for a spatial characterization of the differences across the methods. This analysis was completed with a quantitative assessment *via* error map histogram comparison and the mean absolute dose error per volume histogram to compare different sCT generation methods. Thus, this workflow will be useful in the comparison and localization of errors in the sCT generation method and provides a way forward to sCT quality control within the MRI-based planning RT.

## Data availability statement

The raw data supporting the conclusions of this article will be made available by the authors, without undue reservation.

## Ethics statement

The studies involving human participants were reviewed and approved by Hunter New England Human Research Ethics Committee. The patients/participants provided their written informed consent to participate in this study.

## Author contributions

HC was involved in the study design, code implementation, and data analysis and wrote the manuscript. AB, OA, CL, RC, and J-CN were involved in the study design and reviewed the manuscript. AB also assisted with treatment planning and dose calculation, and OA supported HC for the code implementation and data analysis. ST brought support for the gamma analysis and CC for the statistical analysis. AB-R and MP were in charge of the data delineation. JD and PG were involved in the data collection and critically reviewed the manuscript. All authors contributed to the article and approved the submitted version.

## Funding

This work was partially funded by Region Bretagne (France) through the ARED scholarship program, the University of Rennes 1 “Défis Scientifiques Emergents” grant (France), and a PhD scholarship grant from Australian e-Health Research Centre—CSIRO (Australia).

## Conflict of interest

The authors declare that the research was conducted in the absence of any commercial or financial relationships that could be construed as a potential conflict of interest.

## Publisher’s note

All claims expressed in this article are solely those of the authors and do not necessarily represent those of their affiliated organizations, or those of the publisher, the editors and the reviewers. Any product that may be evaluated in this article, or claim that may be made by its manufacturer, is not guaranteed or endorsed by the publisher.

## Abbreviations

AE, absolute error; APE, absolute percent error; BDM, bulk-density method; CCS, common coordinate system; DLM, deep-learning model; DSC, Dice similarity coefficient; DVH, dose–volume histogram; E, error; ESR, error subregions; GAN, generative adversarial network; MAE, mean absolute error; MAPE, mean absolute percent error; ME, mean error; RSD_AE_, relative standard deviation of absolute error; sCT, synthetic CT; SD, structural description; vMAE, voxel-wise mean absolute error; vMAPE, voxel-wise mean absolute percent error; vME, voxel-wise mean error.
